# 15-month post-COVID syndrome in outpatients: Attributes, risk factors, outcomes, and vaccination status - longitudinal, observational, case-control study

**DOI:** 10.3389/fimmu.2023.1226622

**Published:** 2023-09-12

**Authors:** Max Augustin, Melanie Stecher, Hauke Wüstenberg, Veronica Di Cristanziano, Ute Sandaradura de Silva, Lea Katharina Picard, Elisabeth Pracht, Dominic Rauschning, Henning Gruell, Florian Klein, Christoph Wenisch, Michael Hallek, Philipp Schommers, Clara Lehmann

**Affiliations:** ^1^ Department I of Internal Medicine, Faculty of Medicine and University Hospital Cologne, University of Cologne, Cologne, Germany; ^2^ Center for Molecular Medicine Cologne (CMMC), Faculty of Medicine and University Hospital Cologne, University of Cologne, Cologne, Germany; ^3^ German Center for Infection Research [Deutsches Zentrum für Infektionsforschung (DZIF)], Cologne, Germany; ^4^ Institute of Virology, Faculty of Medicine and University Hospital Cologne, University of Cologne, Cologne, Germany; ^5^ Department IV of Internal Medicine, Klinik Favoriten, Vienna Healthcare Group, Vienna, Austria

**Keywords:** post-COVID syndrome, PCS, long COVID, therapeutic vaccination, symptom clusters, outcome, SARS-CoV-2 IgG, recovery

## Abstract

**Background:**

While the short-term symptoms of post-COVID syndromes (PCS) are well-known, the long-term clinical characteristics, risk factors and outcomes of PCS remain unclear. Moreover, there is ongoing discussion about the effectiveness of post-infection vaccination against severe acute respiratory syndrome coronavirus type 2 (SARS-CoV-2) to aid in PCS recovery.

**Methods:**

In this longitudinal and observational case-control study we aimed at identifying long-term PCS courses and evaluating the effects of post-infection vaccinations on PCS recovery. Individuals with initial mild COVID-19 were followed for a period of 15 months after primary infection. We assessed PCS outcomes, distinct symptom clusters (SC), and SARS-CoV-2 immunoglobulin G (IgG) levels in patients who received SARS-CoV-2 vaccination, as well as those who did not. To identify potential associating factors with PCS, we used binomial regression models and reported the results as odds ratios (OR) with 95% confidence intervals (95%CI).

**Results:**

Out of 958 patients, follow-up data at 15 month after infection was obtained for 222 (23.2%) outpatients. Of those individuals, 36.5% (81/222) and 31.1% (69/222) were identified to have PCS at month 10 and 15, respectively. Fatigue and dyspnea (SC2) rather than anosmia and ageusia (SC1) constituted PCS at month 15. SARS-CoV-2 IgG levels were equally distributed over time among age groups, sex, and absence/presence of PCS. Of the 222 patients, 77.0% (171/222) were vaccinated between 10- and 15-months post-infection, but vaccination did not affect PCS recovery at month 15. 26.3% of unvaccinated and 25.8% of vaccinated outpatients improved from PCS (p= .9646). Baseline headache (SC4) and diarrhoea (SC5) were risk factors for PCS at months 10 and 15 (SC4: OR 1.85 (95%CI 1.04-3.26), p=.0390; SC5: OR 3.27(95%CI 1.54-6.64), p=.0009).

**Conclusion:**

Based on the specific symptoms of PCS our findings show a shift in the pattern of recovery. We found no effect of SARS-CoV-2 vaccination on PCS recovery and recommend further studies to identify predicting biomarkers and targeted PCS therapeutics.

## Introduction

Long-term sequelae after coronavirus disease 2019 (COVID-19) caused by severe acute respiratory syndrome coronavirus 2 (SARS-CoV-2) is a condition which is referred to as post-COVID syndrome (PCS) (1–4). The incidence of PCS after infection with the wild-type Wuhan SARS-CoV-2 variant is estimated with 10–30% of non-hospitalized cases and 50–70% of hospitalized cases (5, 6). PCS is characterized by various clinical manifestations involving several different organ systems, which is partially believed to be due to the wide distribution of the angiotensin converting enzyme 2 (ACE2) receptor that is targeted by SARS-CoV-2 (7). In the context of long-term PCS, this variability in symptom distribution and duration engenders two distinct possibilities, namely, the potential for gradual resolution of symptoms over time or the possibility of worsening and dissemination across distinct organ systems. Several risk factors for PCS have been identified, including female sex, lower SARS-CoV-2 antibody titers, distinct symptom clusters, and a higher number of symptoms during the acute phase (1, 4, 8). While COVID-19 vaccination administered before a SARS-CoV-2 infection may help decrease the risk of developing PCS, there is still debate surrounding the effectiveness of vaccinations given after a SARS-CoV-2 infection for PCS recovery (9–12) (12–14). We established a longitudinal, observational cohort study with a case-control design to identify the long-term outcomes of convalescent patients with and without PCS and evaluate the effects of vaccinations on the course of PCS.

## Methods

### Study design and data collection

We conducted a cohort study on a group of 958 outpatients who had mostly experienced mild symptoms of COVID-19. The study used longitudinal data and followed a case-control design. The participants had received care at the post-COVID outpatient clinic of the University Hospital of Cologne (UHC) between April 6th, 2020 and August 11th 2021 and presented for convalescent plasma donation six weeks after the onset of symptoms. Subsequently, each individual was invited for longitudinal follow-up visits to monitor clinical courses and humoral immune responses following each individuals SARS-CoV-2 infection. As a result, ongoing PCS at month 4 and 7 post-infection was described at the very beginning of the pandemic after mostly mild COVID-19. A more comprehensive overview of the cohort is given in the reference (1).

**Figure 1:**
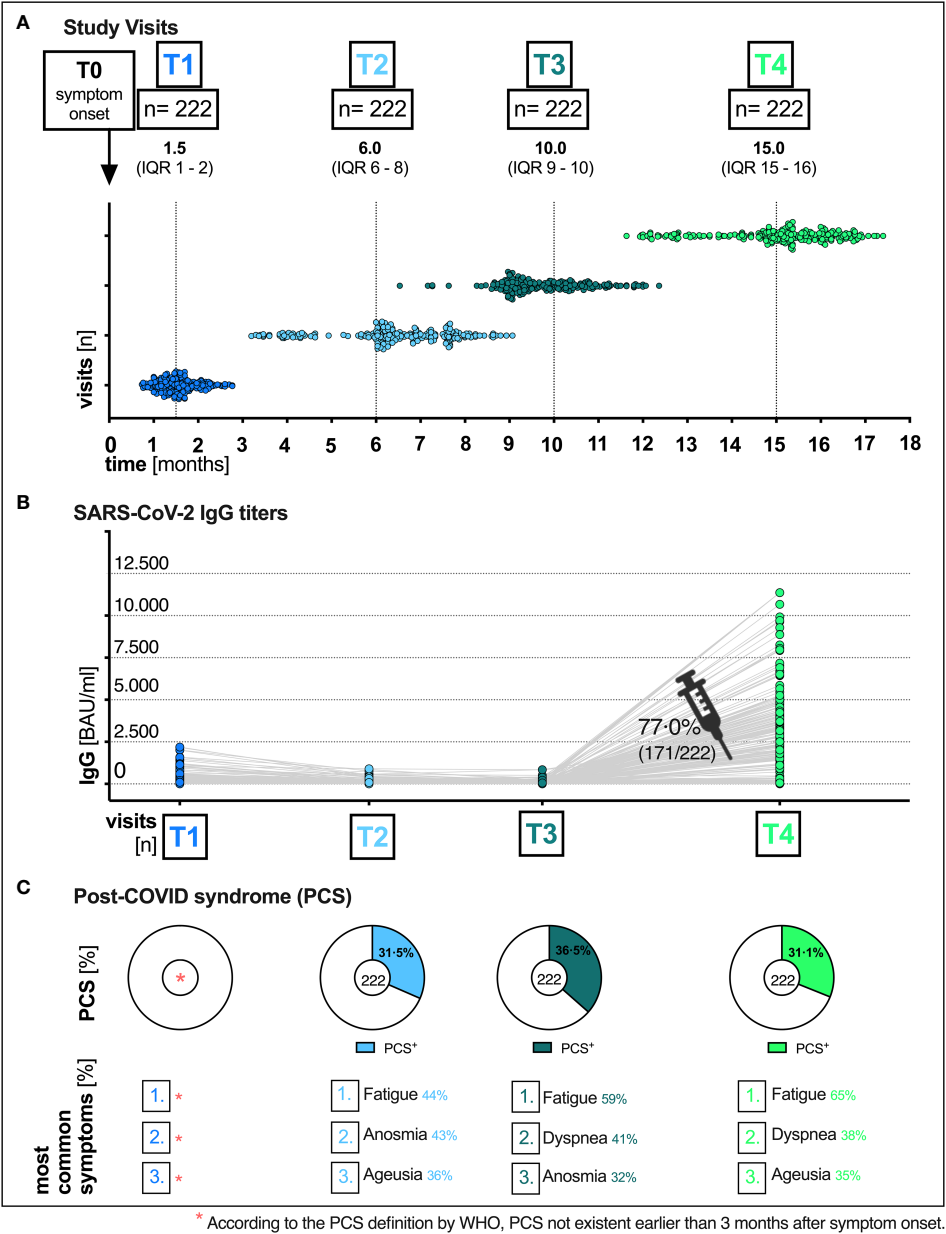
Total visits and humoral immune response of patients at the post-COVID outpatient clinic of the UHC April 6^th^ 2020 till August 11th 2021. **(A)** Median time (months) of the respective visit (T1, T2, T3 and T4) after the onset of symptoms (T0): T1 1.5 (IQR 1–2), T2 6.0 (IQR 6–8), T3 10.0 (IQR 9–10) and T4 15.0 (IQR 15-16). **(B)** SARS-CoV-2 immunoglobulin G (IgG) titers over time, pre and post vaccination. **(C)** Distribution of PCS and the most common symptoms at all visits. UHC, University Hospital Cologne; IQR, interquartile range; T0, symptom onset; T1, first study visit; T2 s study visit; T3, third study visit; T4 fourth study visit; SARS-CoV-2, severe acute respiratory syndrome coronavirus type 2; BAU, binding antibody unit; ml, milliliter.

Out of 958 initial patients, we included 222 (23.2%) outpatients for which follow-up data of 15 months after infection was available. Patients were seen by a clinician over a period of up to 15 months including four scheduled visits, which are defined as T1 (baseline), T2 (6 months), T3 (10 months), and T4 (15 months) in the following. In case of clinical deterioration, patients additionally presented to the UHC as part of unscheduled study visits. At each visit (total visits n=908: scheduled visits n=888, unscheduled visits n=20, **Figure 1**) patients completed a self-reported questionnaire which was evaluated by a trained physician after anamnesis, physical examination and - if needed - symptom-oriented diagnostics such as electrocardiography, pulmonary spirometry, or imaging techniques. In addition, blood samples were collected at all visits.

**Figure 2:**
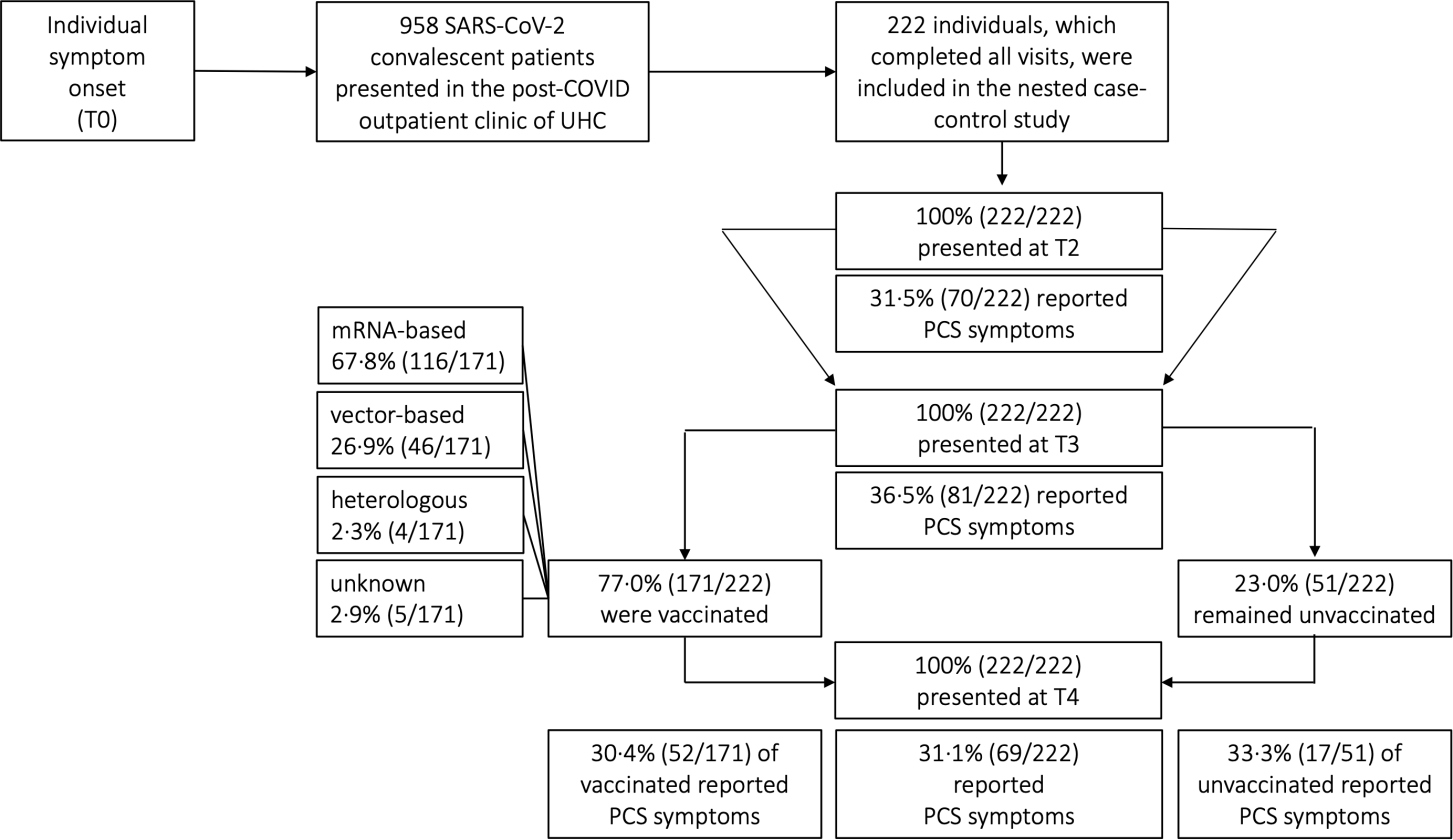
Distribution of post-COVID (PCS) syndrome amongst COVID-19 convalescent patients. UHC, University Hospital Cologne; SARS-CoV-2, severe acute respiratory syndrome coronavirus type 2; COVID-19, coronavirus disease 19; T0, symptom onset; T1, first study visit; T2, second study visit; T3, third study visit; T4, fourth study visit; mRNA, messenger ribonucleic acid.

Cases were defined as patients who presented with long-term PCS at T3 (10 months post-infection) and T4 (15 months post-infection), whereas patients who did not exhibit PCS were considered as controls (**Figure 2**). We estimated the i) probability of PCS in patients who received a COVID-19 vaccination, ii) monitored individual clinical courses and distinct symptom clusters, iii) compared sociodemographic factors, such as age, working status, and iv) compared SARS-CoV-2-specific antibody response in cases and controls. Analyses were stratified according to specific age groups, the vaccination and working status.

### Eligibility criteria and definitions of PCS and symptom clusters (SC)

The following inclusion criteria were specified: (I) age of 18 years and older at the time of diagnosis, (II) diagnosed with SARS-CoV-2 infection using polymerase chain reaction (PCR), and (III) received care at the post-COVID outpatient clinic of the UHC. PCS was defined according to the World Health Organization (WHO) (15) by one or a number of newly emerged symptoms three months after initial SARS-CoV-2 infection such as fatigue, dyspnea, anosmia or ageusia that lasted for at least 2 months and could not be explained by an alternative diagnosis. For detailed analysis of clinical courses, individual symptoms were merged into distinct symptom clusters (SC) based on organ systems, prevalence, and concurrent time of occurrence (SC1 anosmia and ageusia, SC2 fatigue and dyspnea, SC3 sore throat and cough, SC4 headache, SC5 diarrhoea). In other words, most frequent symptoms at baseline (anosmia and ageusia; sore throat and cough) and T4 (fatigue and dyspnea) were merged into distinct clusters based on our previous work (1). In particular, anosmia and ageusia, fatigue and dyspnea and sore throat and cough were stated at the same time in the majority of patients and were thus merged to SC1, SC2 and SC3, respectively. Clusters were classified only if both symptoms were present at the respective time point.

### SARS-CoV-2 vaccinations

Depending on guidelines of national authorities and the local availability of vaccines either a vector-based, or a messenger ribonucleic acid (mRNA)-based, or a heterologous regimen was administered (**Figure 2**). Importantly vaccine regimens were not administered as part of this study but as part of each individual decision following the government vaccination program. Patients were evaluated both before and after receiving vaccination at the UHC outpatient clinic. The query did not specify whether patients were vaccinated as a prophylactic measure against re-infections or for therapeutic treatment of PCS symptoms

### SARS-CoV-2 IgG serology

SARS-CoV-2-specific immunoglobulin G (IgG) titers against the receptor binding domain (RBD) of the SARS-CoV-2 spike protein were measured by the SARS-CoV-2 IgG II Quant assay provided by Abbott on the Alinity i (Abbott, Abbott Park, IL, United States). IgG titers were reported in Binding Antibody Units per milliliter (BAU/ml). According to manufacturer instructions, sample values ≥ 7.1 BAU/ml were interpreted as reactive. Samples with anti-RBD IgG titers above the upper limit of quantification of 11360 BAU/ml were not further diluted.

### Statistical analysis

We performed binomial regression models on the long-term outcome PCS (**Table 1**). The unadjusted odds ratio (OR) with 95% confidence interval (CI) for the probability to develop PCS at T3 (10 months) and T4 (15 months) were reported for defined variables as follows: (i) distinct symptom clusters (SC1-SC5) at baseline, (ii) PCS incidence, (iii) vaccination status, and (iv) SARS-CoV-2 antibody titers. Chi-square tests were performed to identify associations between the different variables and PCS. In addition, metric SARS-CoV-2-specific humoral antibody response (SARS-CoV-2 IgG in BAU/ml) was tested for statistical significance between visits. Normality was assessed by Kolmogorov–Smirnov or Shapiro–Wilk test, respectively. To test for differences in only non-parametrically distributed antibody titers, Kruskal-Wallis tests were performed as applicable. Descriptive analyses for sociodemographic and clinical data are presented as absolute numbers with percentages and median with interquartile range (IQR) as appropriate in **Tables 2** and **S1**. P-values of 0.05 and lower were considered as statistically significant. STATA version 17.0 and GraphPad Prism version 9.4.0 (GraphPad Software, La Jolla, CA) were used to compile the analyses and graph the data.

**Table 1:** Predictors for Post-COVID syndrome (PCS).

	Exposure of variable	Post-COVID syndrome at month 10 (T3), yes (n=81), no (n=141)			Exposure of variable	Post-COVID syndrome at month 15 (T4), yes (n=69), no (n=153)	
		nested case-control design				nested case-control design	
	*n (%)*	*OR (95% CI)*		*p-value*	*n (%)*	*OR (95% CI)*	*p-value*
*Symptom clusters (SC)*							
*Distribution*	*SC at baseline*				*SC at baseline*		
SC1: Anosmia & Ageusia	104 (46.9)	2.04 (1.17 - 3.47)	**0.0114**		104 (46.9)	1.76 (0.97 - 3.12	0.0524
SC2: Fatigue & Dyspneae	1 (0.5)	n.a.	n.a.		1 (0.5)	n.a.	n.a.
SC3: Sore Throat & Cough	60 (27.0)	1.49 (0.83 - 2.76)	0.1972		60 (27.0)	1.70 (0.94 - 3.11)	0.0818
SC4: Headache	122 (55.00)	2.55 (1.43 - 4.62)	**0.0013**		122 (55.00)	1.85 (1.04 - 3.26)	**0.0390**
SC5: Diarrhoea	37 (16.7)	3.33 (1.62 - 6.9)	**0.0007**		37 (16.7)	3.27 (1.54 - 6.64)	**0.0009**
Post-COVID syndrome (PCS)	PCS at 6 months				PCS at 10 months		
	70 (31.5)	17.77 (8.67 - 36.79)	**< 0.0001**		81 (36.5)	41.91 (17.74 - 93.22)	**< 0.0001**
*Vaccination status*	*10 months*				*15 months*		
Vaccinated at	0 (0)	n.a.	n.a.		171 (77.0)	0.87 (0.45 - 1.75)	0.6921
Unvaccinated at 15 months	222 (100)				51 (23.0)		
*SARS-CoV-2 IgG*	*IgG at baseline*				*IgG at baseline*		
*low titers <43 BAU/ml*	56 (25.2)	0.89 (0.48 - 1.65)	0.7303		56 (25.2)	1.24 (0.65 - 2.32)	0.5221

PCS, post-COVID syndrome; SARS-CoV-2, severe acute respiratory syndrome coronavirus type 2; IgG, immunoglobulin G; SC, symptom clusters; OR, odds ratio, 95% CI, 95 percent confidence interval; N, population; n, sample size; IQR, interquartile range; BAU, binding antibody units; ml, millilitre; n.a., not applicable.

Odds ratios (OR) for the development of PCS at month 10 and 15 after symptom onset, respectively.

**Table 2:** Patient characteristics of study population at baseline and 15 month follow-up.

	female (n=136)	male (n=86)	total (N=222)	vaccinated (n=171)	unvaccinated (n=51)
*Age*, *years, median (IQR)*	*49 (36-55)*	*50 (41-59)*	52 (40-59)	*51 (40-57)*	*43 (29-53)*
*n (%)*					
18-39y	39 (32.4)	24 (22.1)	63 (28.4)	39 (32.4)	24 (22.1)
40-49y	33 (17.6)	11 (23.3)	44 (19.8)	33 (17.6)	11 (23.3)
50-59y	60 (33.8)	13 (31.4)	73 (32.9)	60 (33.8)	13 (31.4)
>60y	39 (16.2)	3 (23.3)	42 (18.9)	39 (16.2)	3 (23.3)
*Pre-existing conditions*, *n (%)*	43 (31.6)	26 (30.2)	69 (31.2)	59 (34.5)	10 (19.6)
Malignancies	9 (6.6)	3 (3.5)	12 (5.4)	11 (6.4)	1 (2.0)
Hypertension	11 (8.1)	12 (14.0)	23 (10.4)	20 (11.7)	3 (5.9)
Diabetes	3 (2.2)	2 (2.3)	5 (2.3)	5 (2.9)	0 (0)
Autoimmune disease	5 (3.7)	0 (0)	5 (2.3)	4 (2.3)	1 (2.0)
Chronic lung disease	3 (2.2)	5 (5.8)	8 (3.6)	7 (4.1)	1 (2.0)
WHO grade, n (%)					
I-III	136 (100.0)	86 (100.0)	222 (100.0)	171 (100.0)	51 (100.0)
Symptoms, n (%)					
Cough	96 (70.6)	65 (75.6)	161 (72.5)	125 (73.1)	36 (70.6)
Ageusia	85 (62.5)	45 (52.3)	130 (58.6)	98 (57.3)	32 (62.8)
Anosmia	78 (57.4)	43 (50.0)	121 (54.5)	94 (55.0)	27 (53.0)
Headache	82 (60.9)	40 (46.5)	122 (55.0)	96 (56.1)	26 (51.0)
Muscle and/or body aches	71 (52.2)	44 (51.2)	115 (51.8)	86 (50.3)	29 (56.9)
Fever	51 (37.5)	39 (45.3)	90 (40.5)	67 (39.2)	23 (45.1)
Sore throat	51 (37.5)	25 (29.1)	76 (34.2)	53 (31.0)	23 (45.1)
Diarrhoea	28 (20.6)	9 (10.5)	37 (16.7)	23 (13.4)	14 (27.5)
Fatigue	4 (2.9)	0 (0)	4 (1.8)	4 (2.3)	0 (0)
Dyspneae	2 (1.5)	0 (0)	2 (0.9)	2 (1.2)	0 (0)
No symptoms	1 (0.7)	2 (2.3)	3 (1.4)	2 (1.2)	1 (2.3)
PCS, n (%)					
T1 (1.5 months)	n.a.	n.a	n.a.	n.a.	n.a
T2 (6 months	54 (39.7)	16 (18.6)	70 (31.5)	57 (33.3)	13 (25.5)
T3 (10 months)	63 (46.3)	18 (20.9)	81 (36.5)	62 (36.3)	19 (37.3)
T4 (15 months)	52 (38.2)	17 (19.8)	69 (31.1)	52 (30.4)	17 (33.3)
Symptom clusters (SC)					
Distribution at baseline, n (%)					
* *SC1: Anosmia & Ageusia	68 (50.0)	36 (41.9)	104 (46.9)	80 (46.8)	24 (47.1)
* *SC2: Fatigue & Dyspneae	1 (0.7)	0 (0)	1 (0.5)	1 (0.6)	0 (0)
* *SC3: Sore Throat & Cough	40 (29.4)	20 (23.3)	60 (27.0)	42 (24.6)	18 (35.3)
* *SC4: Headache	82 (60.3)	40 (46.5)	122 (55.0)	96 (56.1)	26 (51.0)
* *SC5: Diarrhoea	28 (20.6)	9 (10.5)	37 (16.7)	23 (13.5)	14 (26.4)
Distribution at month 15, n (%)					
* *SC1: Anosmia & Ageusia	1 (0.7)	0 (0)	1 (0.5)	0 (0)	1 (2.3)
* *SC2: Fatigue & Dyspneae	16 (11.8)	1 (1.1)	17 (7.7)	12 (7.0)	5 (9.8)
* *SC3: Sore Throat & Cough	0 (0)	0 (0)	0 (0)	0 (0)	0 (0)
* *SC4: Headache	4 (2.9)	0 (0)	4 (1.8)	2 (1.2)	2 (3.9)
* *SC5: Diarrhoea	12 (8.8)	4 (4.5)	16 (7.2)	12 (7.0)	4 (7.8)
Vaccination status, n (%)					
Vaccinated at 15 months	107 (78.7)	64 (74.2)	171 (77.0)	171 (100.0)	0 (0)
Unvaccinated at 15 months	29 (21.3)	22 (25.8)	51 (23.0)	0 (0)	51 (100.0)
Working status at 15 months, n (%)					
Working again	99 (69.9)	68 (79.1)	167 (75.2)	125 (73.1)	42 (82.4)
Sick leave	20 (14.7)	13 (15.1)	33 (14.9)	29 (17.0)	4 (7.8)
Unknown	17 (4.4)	5 (5.8)	22 (9.9)	17 (9.9)	5 (9.8)

PCS, post-COVID syndrome; WHO, world health organisation; N, population; n, sample size; IQR, interquartile range; y, years; n.a., not applicable.

### Ethical considerations

The study received ethical approvals from the ethics committee of the UHC reference number: 16_054 and 20-1187_1.

## Results

### Characteristics of the study population

A total of 222 patients with PCR-confirmed SARS-CoV-2 infection who received follow-up care at the outpatient clinic of the UHC between April 6th 2020 and August 11th 2021 were included in the analyses. The majority of patients included in our study were women [61.3% (136/222)]. The median age of all patients was 52 years (IQR 40-59) (**Table 2**). Most patients were healthy and did not have any pre-existing comorbidities (153/222; 68.9%). Of those that did, the main pre-existing conditions were hypertension (23/222; 10.4%), malignancies (12/222; 5.4%) and chronic lung disease (8/222; 3.6%). More detailed sociodemographic and clinical characteristics are listed in **Table 2**. Since our study cohort exhibited disease symptoms between February and May 2020, it is most probable that the SARS-CoV-2 infections of our cohort were caused by the wild-type variant of Wuhan. No patient had received any specific therapy during his initial COVID-19. All patients had a mild course of COVID-19 in the outpatient setting (WHO disease grade I-III (16); 100.0%, 222/222, **Table 2**). Patients were followed up for a median time of 15 months (interquartile range (IQR) 15-16, (**Figure 1A**). Among all patients, 77.0% (171/222) have received at least one vaccination during the observation period between 10 and 15 months after symptom onset. 59.6% (102/171) and 40.4% (69/171) of vaccinated patients received single and double vaccination, respectively. Vaccinations were administered at a median of 60 days (IQR 31-84) prior to 15 months visit. Messenger ribonucleic acid (mRNA)-based vaccination regimens (116/171; 67.8%) were the most used (**Figure 2**), followed by vector-based (46/171; 26.9%) and heterologous (4/171; 2.3%) regimens. In 2.9% (5/171) of patients, the type of vaccination was unknown. While baseline symptoms of vaccinated and unvaccinated patients did not differ, vaccinated patients stated to have more pre-existing conditions (34.5% versus 19.6%, **Table 2**). At 6, 10 and 15 months after symptom onset 14.9% (33/222), 15.3% (34/222) and 14.9% (33/222) of patients were on sick leave, respectively (**Tables 2**, **S3**).

### Distinct baseline and long-term symptom clusters

**Figure 3:**
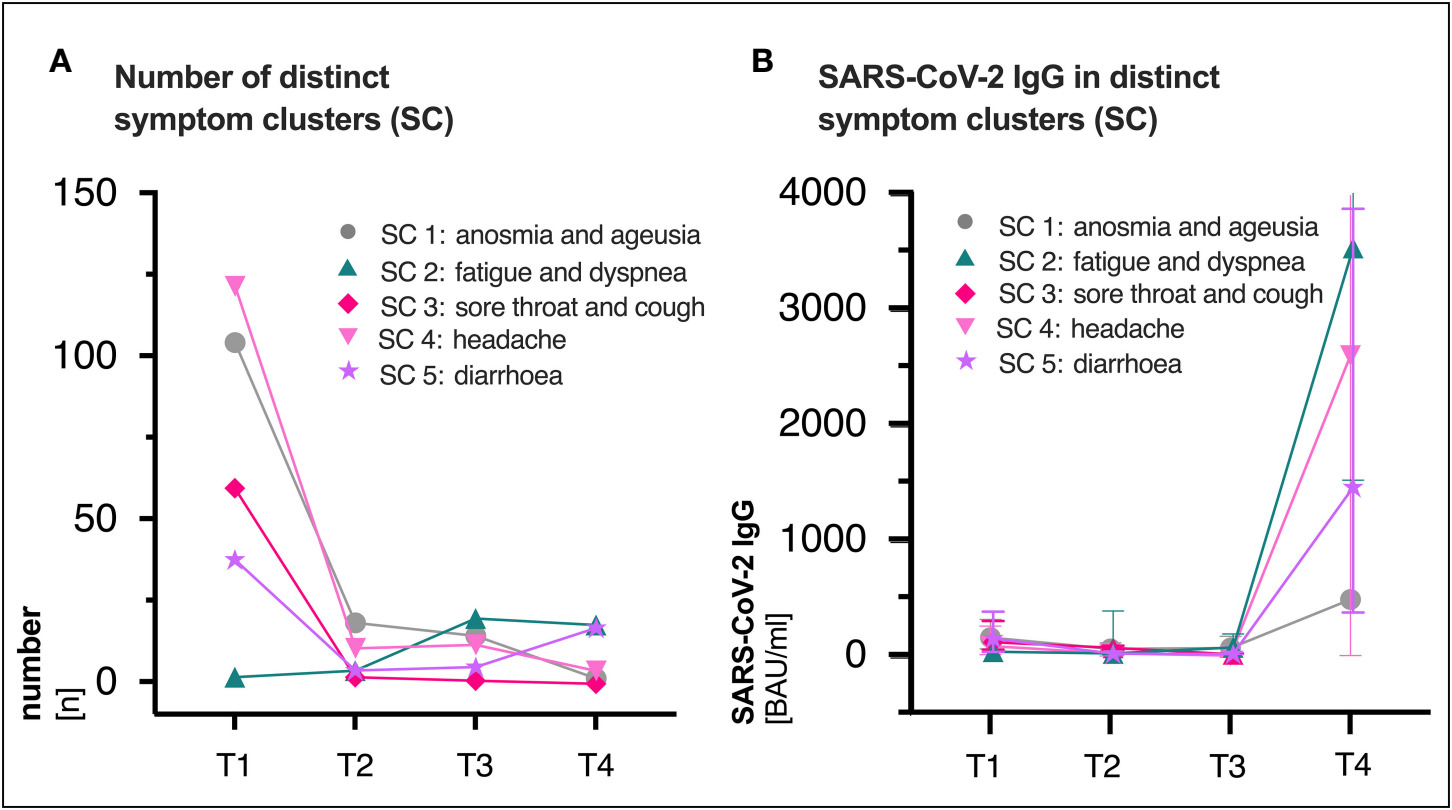
**(A)** Distribution of distinct symptom clusters (SC) at the respective visit (T1, T2, T3 and T4). **(B)** Median (IQR) SARS-CoV-2 immunoglobulin G (IgG) titers (BAU/ml) in distinct clusters over time. SC3 was not existent at T4 (n=0), thus no antibody titers were available. SARS-CoV-2, severe acute respiratory syndrome coronavirus type 2; BAU, binding antibody protein; IQR, interquartile range; ml, milliliter; T1, first study visit; T2, second study visit; T3, third study visit; T4, fourth study visit.

The most common reported COVID-19 related symptoms at baseline (**Table 2**) were cough (72.5; 161/222), change in taste or smell (58.6%; 130/222 and 54.5%; 121/222 respectively), headache (55.0%; 122/222) as well as muscle and/or body aches (51.8%; 115/222). A total of 31.1% (69/222) patients reported symptoms consistent with PCS after a median time of 15 (IQR 15-16) months after infection (**Figures 1C**, **2**, **Table 2**). Similar rates of PCS (31.5%; 70/222 and 36.5%; 81/222) were found after a median time of 6 (IQR 6-8) or 10 months (IQR 15-16), respectively (**Figure 1C**, **Table 2**). Among patients with a PCS at T4 (**Table S1**), the most frequent symptoms (**Figure 1C**) after 15 months were fatigue (65.2%; 45/69), dyspnea (37.7%; 26/69), ageusia (34.8%; 24/69) and diarrhoea (23.2%; 16/69). Over time, the frequencies and characteristics of the symptoms changed. Based on the clinical presentation of patients, symptoms were assigned to five distinct symptom clusters (SC1-SC5) (**Figure 3**, **Table 2**). Comparison of SC1-SC5 at each visit revealed distinct clinical manifestations in early and long-term disease courses, respectively. While clusters such as anosmia and ageusia (SC1), sore throat and cough (SC3) and headache (SC4) were most common at 6 weeks after symptom onset (SC1: n=104, SC3: n=60 and SC4: n=122), they were least present at month 15 (SC1: n=1, SC3: n=0 and SC4: n=4). In contrast, the prevalence of fatigue and dyspnea (SC2) was low in the beginning (SC2: n=1) and increased over time (SC2: n=17). Only the frequency of diarrhoea (SC5) showed two peaks: at first and at last visit (T1: n=37, T4: n=16, **Figure 3A**).

### Similar distribution of SARS-CoV-2 immunoglobulin G (IgG) regardless of clinical characteristics

While SARS-CoV-2 RBD immunoglobulin G (IgG) titers gradually declined from T1 to T3, IgG levels at T4 increased in all patients after vaccination (median BAU/ml (IQR); T1: 111 (43-284), T2: 51 (21-114), T3: 40 (15-94), T4: 2308 (320-4334), **Figure 1B**). **Figure S1** shows SARS-CoV-2 IgG titers (BAU/ml) stratified by (A) presence/absence of (PCS^+^ versus PCS^−^), (B) sex (female versus male) and (C) age (18-59 versus 60-79), respectively. Pre-vaccination SARS-CoV-2 IgG titers were comparably distributed across visits when comparing patients with and without PCS, female, and male patients or younger (18-59 years) and older (60-79 years) patients (**Figure S1**, **Table S2**). After vaccination between T3 and T4, significantly increased titers (p <.0001 for all comparisons) were observed in all three stratified groups. (PCS^+^ p <.0001; PCS^−^ p <.0001); female p <.0001; male p <.0001; age 18-59 p <.0001; age 60-79 p <.0001, **Figures S1A–C**, **Table S2**). Interestingly, antibody titers (BAU/ml) were distributed differently in symptom clusters SC1-SC5, with SC2 and SC4 having the highest and SC1 and SC5 having the lowest levels of SARS-CoV-2 IgG (**Figure 3B**). However, no significant differences were observed.

### PCS recovery after 15 months in patients with and without SARS-CoV-2 vaccination

**Figure 4:**
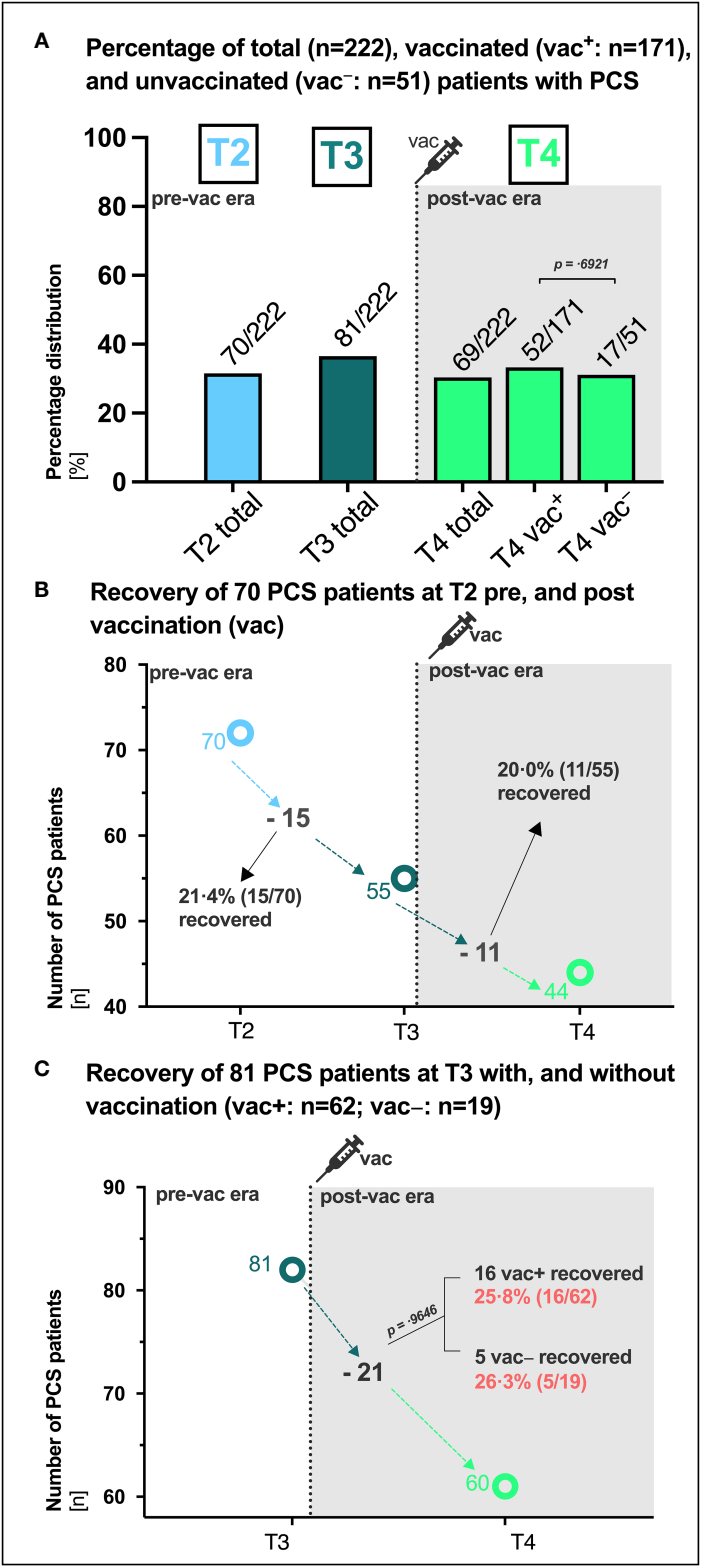
PCS in vaccinated and unvaccinated SARS-CoV-2 convalescents. **(A)** Absolute (n) and percentage (%) distribution of patients with post-COVID syndrome (PCS) at the respective visit (T1, T2, T3, T4). **(B)** Recovery of 70 PCS patients at T2 between T2 and T3 (ΔT2/T3) pre vaccination and between T3 and T4 (ΔT3/T4) post vaccination, respectively. **(C)** Recovery of 81 PCS patients at T3 with (vac^+^) and without (vac^−^) vaccination (vac). P-values derived from Chi-square tests. P-values of 0.05 and lower were considered as statistically significant. vac, vaccination.

Patients were stratified based on their vaccination status at last visit [vac^+^: 77.0% (171/222), vac^−^: 23,0% (51/222)], and the presence of PCS was assessed over a total period of 15 months (**Figure 4A**). The percentage of patients with PCS was overall comparable at the different time points and did not appear to be affected by vaccination (vac^+^ versus vac^−^: T4 30.4% (52/171) versus 33.3% (17/51), p= .6921 **Figure 4A**). To further evaluate if vaccination can influence PCS recovery, we assessed the clinical courses and intraindividual outcomes over time of the 70 patients that were identified with PCS at T2 (6 months, **Figure 4B**). Regardless of whether vaccinated or not, 21.4% (15/70) of PCS patients recovered prior to vaccination between T2 and T3 and 20.0% (11/55) of PCS patients recovered after vaccination between T3 and T4 (**Figure 4B**). In addition, intraindividual outcomes of the 81 patients with PCS at T3 were also assessed (**Figure 4C**). Here, 25.8% (16/62) of vaccinated and 26.3% (5/19) of unvaccinated PCS patients recovered until T4 (p =.9646). In addition, according to the unadjusted binomial regression model (**Table 1**) there was no significant difference in the probability of experiencing a PCS after 15 months between those who were vaccinated and those who were not (OR 0.87; 95% CI: 0.45 1.75; p =0.6921).

### Diarrhoea and headache at baseline were predictors for PCS at months 10 and 15, respectively

Unadjusted binomial logistic regression models revealed several symptoms during acute COVID-19 that were associated with an increased risk of PCS after 10 and 15 months, respectively (**Table 1**): Diarrhoea (10 months: OR 3.33; 95%CI 1.62 6.9, p =.0007; 15 months: OR 3.27; 95%CI 1.54 6.64, p =.0009) and headache (10 months: OR 2.55; 95%CI 1.43 4.62, p =.0013; 15 months: OR 1.85; 95%CI 1.04 3.26, p =.0390) were risk factors for PCS at T3 and T4, respectively. Moreover, as expected, presence of PCS strongly increased the risk of PCS at the following visit (10 months: OR 17.77; 95%CI 8.67 36.79, p <.0001; 15 months: OR 41.91; 95%CI 17.74 93.22, p <.0001, **Table 1**).

## Discussion

Given the persistent lack of objective diagnostic criteria and biomarkers, a reliable clinical diagnosis of PCS requires a detailed knowledge of its long-term clinical course. In our longitudinal and observational cohort study, we described the recovery from initially unvaccinated PCS patients, most likely infected with the wild-type SARS-CoV-2 variant of Wuhan in early 2020, with and without vaccination and humoral immune responses at the outpatient clinic of the UHC, Germany. In line with our previous analysis of our mild COVID-19 outpatient cohort, which found a 34.8% prevalence at month 7 post-infection (1), 36.5% and 31.1% of patients in this cohort had PCS at month 10 and 15, respectively (**Figures 1C**, **2**, **Table 2**). This prevalence of long-term PCS in about one third of patients after mild COVID-19 is within the 7.5% to 41% PCS estimates derived from large meta-analyses (17–19). In detail, distinct symptom clusters in this study such as anosmia and ageusia (SC1), sore throat and cough (SC3), and headache (SC4) decreased longitudinally (**Figure 3**). Persistent anosmia and ageusia due to olfactory neuroepithelial damage is well documented (20–22), and our findings are consistent with these studies. Specifically, we observed that 6% of patients still experienced anosmia/ageusia at month 10, which is in line with the reported rates at month 6 and 12. However, after 15 months, 99.5% of patients had recovered from these symptoms, suggesting that they likely have minimal long-term impact on PCS (**Figure 3**). It is worth noting that unlike PCS patients who were initially hospitalized, our study focused on outpatients only. As such, we found that the rates of sore throat, cough, and headache were low at month 15, which differs from the experiences reported by previously hospitalized PCS patients (23).

In contrast, the frequency of fatigue and dyspnea (SC2) and diarrhoea (SC5) plateaued and remained dominant in the long-term PCS (**Figure 3**). The 15-month persistence of fatigue and dyspnea in 24.6% of PCS patients in this cohort (**Table S1**) agrees to current literature. It has been observed that fatigue and dyspnea are among the most common symptoms experienced by individuals with long-term PCS (1, 3, 23). Moreover, our observations align with previous research regarding the prevalence of gastrointestinal symptoms, including diarrhoea. Studies such as the one by (24) and (23) have reported similar patterns, with 9.6% and 5.5% of initially hospitalized patients experiencing gastrointestinal symptoms at the 6-month and 15-month post-infection stages, respectively. In line with these trends, our own study indicates a significant prominence of diarrhoea, with rates of 7% in patients and 23% in PCS patients at the 15-month point (**Table S1**). Furthermore, our findings support the role of the SARS-CoV-2 tissue reservoirs in the gut in the PCS pathogenesis, as indicated by the fact that diarrhoea at baseline significantly increased the risk of developing PCS at both month 10 and 15 (**Table 1**), which is in line with our previous analysis (1). Other clinical PCS risk factors in this study were headache and presence of PCS itself (**Table 1**), suggesting that ongoing assessment of clinical features is essential for improving the care of PCS patients. Unlike our previous analyses (1), low antibody titers at baseline were not associated with the risk of long-term PCS at month 10 or 15 (**Table 1**). The reason for this discrepancy could be attributed to the fact that the current study measured IgG specific to the receptor-binding domain (RBD), whereas the first study analysed SARS-CoV-2 IgG reactive to the complete S1 domain of the spike protein (PS1). Besides, in contrast to lower PS1 IgG levels in long-term PCS patients (25), humoral SARS-CoV-2 IgG titers were equally distributed over time by age groups, sex, and presence/absence of PCS until month 15 (**Figure S1**, **Table S2**). An analysis of 65 unvaccinated Post-COVID Syndrome (PCS) in- and outpatients conducted three months post-infection showed contradictory findings: PCS patients aged 40 years or older exhibited significantly higher antibody titers, as did those aged below 40 years (26). Similarly, our study found an equitable distribution of SARS-CoV-2 IgG between male and female PCS patients (26). When stratified by distinct symptom clusters (SC) at month 15, SARS-CoV-2 IgG levels showed more pronounced differences (SC2: fatigue and dyspnea > SC3: sore throat and cough > SC5: diarrhoea > SC4: headache, **Figure 3B**). However, statistical significance could not be established due to limited number of cases.

Another unanswered question is whether COVID-19 vaccination after SARS-CoV-2-infection can help improve symptoms in cases where PCS has developed. In this study, recovery of PCS proceeded similarly in 21.4% of PCS patients pre-vaccination and in 20.0% of PCS patients post-vaccination (**Figure 4B**) as well as in 25.8% of PCS patients with and 26.3% without SARS-CoV-2 vaccination (**Figure 4C**). This implies that PCS persisted in about 63% of patients with pre-existing PCS at T2 and 74% of patients at T3, regardless of vaccination status. Around 30.4% of vaccinated patients and 33.3% of unvaccinated patients still had PCS at month 15 (**Figures 2**, **4A**). In contrast to the 66.7% vaccination coverage of the general population in Germany in summer/autumn 2021 (27), 77.0% of patients in this study received at least one vaccination in median 60 days before the final assessment at month 15. Consistent with a 64-fold surge in SARS-CoV-2 IgG (BAU/ml) observed in a small cohort of 42 PCS outpatients three weeks after a single vaccination (28), our study noted a 58-fold increase in antibody titers among PCS patients ten weeks post-vaccination. Interestingly, in line with prior data (29), the RBD-specific SARS-CoV-2 IgG showed uniform distribution between vaccinated PCS^+^ and PCS^−^ patients (**Figure S1**). While a significant increase in RBD-specific SARS-CoV-2 IgG of vaccinated patients in our cohort was observed after a median of 60 days (**Figures 1B**, **S1**), the period might have been too short to notice any clinical improvement. Moreover, 60% of patients in our cohort received only a single shot before the last visit, which has been suggested to be inferior to double administration in some studies (30, 31). Other studies found that SARS-CoV-2 vaccination after initial SARS-CoV-2 infection had temporary beneficial effect on clinical improvement or PCS recovery (29, 32). However, in some studies, therapeutic double and single vaccination produced a temporary beneficial effect on some PCS symptoms lasting between 21 and 67 days (10, 33). Of particular note, early administration of vaccines may be more effective than the late administration (34). In contrast, it is important to separate therapeutic vaccination from preventive vaccination against SARS-CoV-2. Administered vaccinations prior to SARS-CoV-2 infections are associated with lower PCS prevalence in 45% (35), 41% (10), 51% (9) and 15% (36) of cases. However, our patient cohort had not received preventive vaccination and, coupled with the fact that they were likely infected with the Wuhan wild type, this may be a factor contributing to the high proportion of PCS observed in over 36.5% and 31.1% of patients at month 10 and 15, respectively.

When dealing with long-term PCS, it is particularly important to discuss whether the persistent symptoms are a consequence of the viral disease in the biological context or of the pandemic. In fact, pandemic-related circumstances such as infection-control measures lead to restrictions in everyday life (37) that can affect physical and mental health in SARS-CoV-2 positive (38, 39) as well as uninfected individuals (40–43). Given various factors such as distinct SARS-CoV-2 variants, vaccination status prior to infection and pandemic circumstances, the true prevalence of PCS may be lower than reported in certain circumstances. For instance, in a British study with over a million participants, PCS was described in anywhere between 7.8% to 17% of patients 3 months post-infection (44). However, the wide range of PCS prevalence reported in different studies and the difficulty in comparing results are attributable to multiple factors, such as variations in sampling size, selection criteria, follow-up duration, and symptom assessment methods. Moreover, uncontrolled studies such as ours combined with various stressful pandemic circumstances run the risk of overreporting of PCS prevalence (45). Besides that, it is worth acknowledging the potential significance of SARS-CoV-2 reinfections in relation to the prevalence of PCS. However, it is important to note that our study was conducted during the initial phases of the COVID-19 pandemic, characterized by stringent infection-control measures and limited viral evolution. Also, the increase in RBD-specific antibody titers was observed only following the administration of SARS-CoV-2 vaccinations between time points T3 and T4. Consequently, we believe that the potential impact of reinfections was minimal within the scope of our study.

Our study has further limitations that should be considered in interpreting our findings. Firstly, due to the limited number of patients included in our study, the unadjusted odds ratios for most of the reported symptoms were definite but not statistically significant, resulting in a loss of statistical power. Secondly, we were unable to conduct subgroup analyses to assess the outcomes of mRNA-based or vector-based vaccination regimens due to the limited case numbers. However, we did provide baseline characteristics of subgroups stratified by sex or vaccination status, which may be informative. In summary, our data indicate that distinct symptom clusters undergo significant changes over time, from baseline to 15 months post-infection. Initially insignificant symptoms become dominant in long-term PCS, and vice versa. However, SARS-CoV-2-specific IgG titers were equally distributed across age groups, sex and PCS over time. Interestingly, in our cohort, diarrhoea, headache, and PCS itself significantly increased the risk of PCS at month 15, while recovery from PCS was found to be similar, whether the individuals were vaccinated or not. Ultimately, our results underscore the importance of continued clinical monitoring of PCS patients and the urgency for developing targeted therapeutic interventions for this population.

## Data availability statement

The raw data supporting the conclusions of this article will be made available by the authors, without undue reservation.

## Ethics statement

The studies involving humans were approved by The study received ethical approvals from the ethics committee of the University Hospital Cologne reference number: 16_054 and 20-1187_1. The studies were conducted in accordance with the local legislation and institutional requirements. The participants provided their written informed consent to participate in this study.

## Author contributions

MA, MS, PS, and CL: conceptualization, methodology, investigation, data curation, and writing—original draft preparation. MA, MS, PS and CL: software and validation. MA, MS, HW, VC, US, PS and CL: formal analysis. MA, HW, VC, EP, DR, HG, FK, FK, MH, PS and CL: resources. MA, MS, HW, VC, US, LP, EP, DR, HG, FK, CW, MH, PS and CL: writing—review and editing. MA, MS, PS and CL: visualization. CL, PS, FK, and MH: supervision. CL and MA: project administration. MA, CL, PS and MH: funding acquisition. All authors contributed to the article and approved the submitted version.
