# Supplementary material for: 15-month post-COVID syndrome in outpatients: Attributes, risk factors, outcomes, and vaccination status - longitudinal, observational, case-control study
Source: Front Immunol. 2023 Sep 12;14:1226622. doi: 10.3389/fimmu.2023.1226622 (PMC10540070; doi:10.3389/fimmu.2023.1226622)
Supplement: Supplementary file 1 [file DataSheet_1.pdf]

Supplementary Material

Attributes, risk factors and outcomes of 15-month post-COVID syndrome in outpatients with and without SARS-CoV-2 vaccination: Results from a longitudinal, observational cohort study with case-control design

Max Augustin [1,2,3,†] and Melanie Stecher [1,3†], Hauke Wüstenberg [1], Veronica di Cristanziano [4], Ute Sandaradura de Silva [1,2,3], Elisabeth Pracht [1], Dominic Rauschning [1], Henning Gruell [4], Florian Klein [2,4], Christoph Wenisch [5], Michael Hallek [1], Philipp Schommers [1,2,3,4 *] and Clara Lehmann [1,2,3 *]

*** Correspondence:** Clara Lehmann, clara.lehmann@uk-koeln.de

Supplementary Figures

Supplementary Figure 1

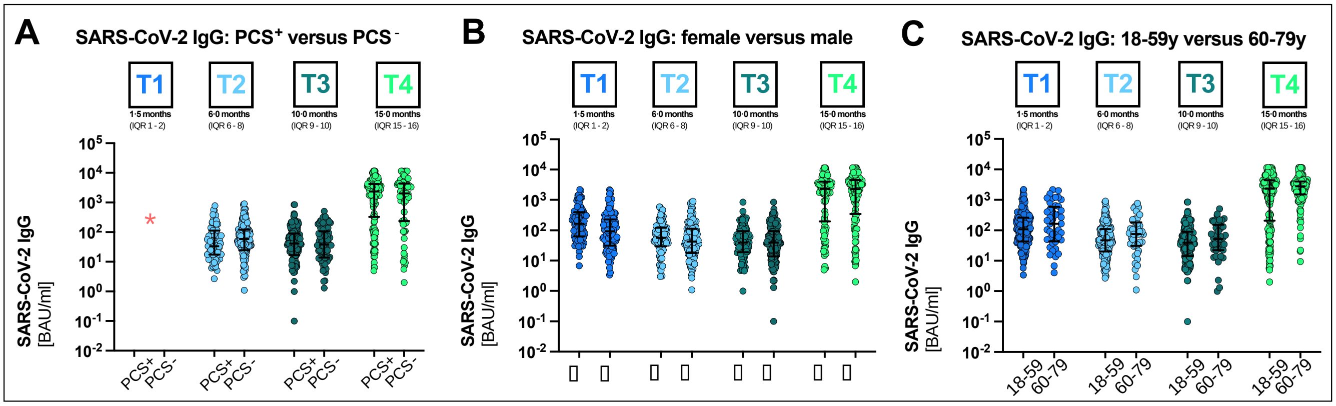

* According to the PCS definition by WHO, PCS not existent earlier than 3 months after symptom onset.

Figure S1. Stratified SARS-CoV-2 immunoglobulin G (IgG) titers (median (IQR) BAU/ml) based on **A** presence/absence of post-COVID syndrome (PCS), **B** sex and **C** age. PCS+, patients with PCS; PCS−, patients without PCS; SARS-CoV-2, severe acute respiratory syndrome coronavirus type 2; IQR, interquartile range; BAU, binding antibody units; ml, milliliter; y, years.

Supplementary Tables

Supplementary Table 1

	female (n=52)	male (n=17)	total (N=69)
Age, *years, median (IQR)*	*50 (34-57)*	*56 (47-63)*	52 (38-60)
Pre-existing conditions, *n (%)*	19 (36·5)	4 (23·5)	23 (33·3)
Malignancies	5 (9·6)	0	5 (7·2)
Hypertension	5 (9·6)	2 (11·8)	7 (7·2)
Diabetes	2 (3·8)	2 (11·8)	4 (5·8)
Autoimmune disease	2 (3·8)	0	2 (2·9)
Chronic lung disease	1 (1·9)	2 (11·8)	3 (4·3)
WHO grade, *n (%)*			
I-III	52 (100·0)	17 (100·0)	69 (100·0)
Symptoms, *n (%)*			
Fatigue	35 (67·3)	10 (58·8)	45 (65·2)
Dyspneae	23 (44·2)	3 (17·6)	26 (37·7)
Ageusia	16 (30·8)	8 (47·1)	24 (34·8)
Diarrhoea	11 (20·4)	5 (29·4)	16 (23·2)
Muscle and/or body aches	9 (18·5)	1 (5·9)	10 (14·5)
Anosmia	8 (16·7)	0 (0)	8 (11·6)
Fever	2 (3·8)	2 (11·8)	4 (5·8)
Headache	3 (5·6)	1 (5·9)	4 (5·8)
Sore throat	1 (1·9)	1 (5·9)	2 (2·9)
Cough	1 (1·9)	0 (0)	1 (1·4)
No symptoms	0 (0)	0 (0)	0 (0)
Distribution at month 15, *n (%)*			
SC1: Anosmia & Ageusia	1 (1·9)	0 (0)	1 (0·4)
SC2: Fatigue & Dyspneae	16 (30·8)	1 (5·9)	17 (24·6)
SC3: Sore Throat & Cough	0 (0)	0 (0)	0 (0)
SC4: Headache	4 (7·7)	0 (0)	4 (5·8)
SC5: Diarrhoea	12 (8·7)	4 (23·5)	16 (23·2)
Vaccination status*, n (%)*			
Vaccinated at 15 months	42 (80·8)	10 (58·8)	52 (75·4)
Unvaccinated at 15 months	10 (19·2)	7 (41·2)	17 (24·6)
Working status at 15 months, *n (%)*			
Working again	*40 (76·9)*	*11 (64·7)*	51 (73·9)
Sick leave	*7 (13·5)*	*4 (23·5)*	11 (15·9)
Unknown	*5 (9·6)*	*2 (11·8)*	7 (10·1)

Table S1. Detailed patient characteristics of 69 PCS patients 15 months post-infection (T4). PCS, post-COVID syndrome; WHO, world health organisation; T4, last study visit; N, population; n, sample size; IQR, interquartile range; y, years; n.a., not applicable.

Supplementary Table 2

	SARS-CoV-2 IgG titers [BAU/ml median (IQR)]				
	T1	T2	T3	T4	p-value vac$^+$ vs. vac$^-$
PCS					
PCS$^+$	107 (43-278)	34 (18-113)	38 (14-113)	2015 (234-4344)	**<0·0001**
PCS$^-$	113 (41-320)	59 (25-121)	41 (17-91)	2351 (327-4235)	
Age					
18-59 years	108 (43-260)	47 (20-110)	39 (14-90)	2308 (207-4432)	**<0·0001**
60-79 years	163 (44-588)	76 (30-181)	51 (22-152)	2797 (1521-4001)	
Sex					
female	163 (53-396)	57 (30-121)	40 (19-95)	2322 (197-4013)	**<0·0001**
male	94 (31-230)	42 (18-113)	40 (14-95)	2293 (349-4545)	
**Symptom clusters (SC)**					
SC1: Anosmia & Ageusia	145 (48-304)	50 (20-100)	55 (17-157)	479 (479-479)	>0·05
SC2: Fatigue & Dyspneae	33 (33-33)	20 (7-389)	71 (18-198)	3528 (1519-5056)	**<0·0001**
SC3: Sore Throat & Cough	107 (43-289)	57 (38-75)	1 (1-1)	n.a	n.a.
SC4: Headache	107 (32-278)	43 (21-113)	23 (9-43)	2618 (22-9814)	>0·05
SC5: Diarrhoea	152 (33-383)	21 (0-37)	14 (12-30)	1459 (374-3871)	**<0·0001**

Table S2. Distribution of SARS-CoV-2 IgG titers (BAU/ml) in stratified groups. Data information: For statistical analysis, non-parametric Kruskal-Wallis tests with Dunn's multiple comparisons were used. p <0·05 shows statistical significance. PCS, post-COVID syndrome; SARS-CoV-2, severe acute respiratory syndrome coronavirus type 2; IgG, immunoglobulin G; BAU, binding antibody units; ml, milliliter; vac$^+$, vaccinated; vs., versus; vac$^-$, unvaccinated; N, population; n, sample size; IQR, interquartile range; n.a., not applicable

Supplementary Table 3

	female (n=136)	male (n=86)	total (N=222)	vaccinated (n=171)	unvaccinated (n=51)
Working status at 6 months, *n (%)*					
Working again	*101 (74.3)*	*59 (68.6)*	*160 (72.0)*	*126 (73.7)*	*34 (66.7)*
Sick leave	*16 (11.8)*	*17 (19.8)*	*33 (14.9)*	*25 (14.6)*	*8 (15.7)*
Unknown	*19 (14.0)*	*10 (11.6)*	*29 (13.1)*	*20 (11.7)*	*9 (17.6)*
Working status at 10 months, *n (%)*					
Working again	*103 (75.7)*	*57 (66.3)*	*160 (72.1)*	*120 (70.2)*	*40 (78.4)*
Sick leave	*20 (14.7)*	*14 (16.3)*	*34 (15.3)*	*23 (13.5)*	*11 (21.6)*
Unknown	*13 (9.6)*	*15 (17.4)*	*28 (21.6)*	*28 (16.4)*	*0 (0)*
Working status at 15 months, *n (%)*					
Working again	*99 (69.9)*	*68 (79.1)*	*167 (75.2)*	*125 (73.1)*	*42 (82.4)*
Sick leave	*20 (14.7)*	*13 (15.1)*	*33 (14.9)*	*29 (17.0)*	*4 (7.8)*
Unknown	*17 (4.4)*	*5 (5.8)*	*22 (9.9)*	*17 (9.9)*	*5 (9.8)*

Table S3. Working status of PCS patients at 6-, 10- and 15 months post-infection. PCS, post-COVID syndrome; n, sample size; %, percentage.
